# Maximal Expression of *Foxl2* in Pituitary Gonadotropes Requires Ovarian Hormones

**DOI:** 10.1371/journal.pone.0126527

**Published:** 2015-05-08

**Authors:** Maria K. Herndon, John H. Nilson

**Affiliations:** School of Molecular Biosciences, Washington State University, Pullman, Washington, United States of America; Florida International University, UNITED STATES

## Abstract

Gonadotropin-releasing hormone (GnRH) and activin regulate synthesis of FSH and ultimately fertility. Recent *in vivo* studies cast SMAD4 and FOXL2 as master transcriptional mediators of activin signaling that act together and independently of GnRH to regulate *Fshb* gene expression and female fertility. Ovarian hormones regulate GnRH and its receptor (GNRHR) through negative and positive feedback loops. In contrast, the role of ovarian hormones in regulating activin, activin receptors, and components of the activin signaling pathway, including SMAD4 and FOXL2, remains understudied. The widespread distribution of activin and many of its signaling intermediates complicates analysis of the effects of ovarian hormones on their synthesis in gonadotropes, one of five pituitary cell types. We circumvented this complication by using a transgenic model that allows isolation of polyribosomes selectively from gonadotropes of intact females and ovariectomized females treated with or without a GnRH antagonist. This paradigm allows assessment of ovarian hormonal feedback and distinguishes responses that are either independent or dependent on GnRH. Surprisingly, our results indicate that *Foxl2* levels in gonadotropes decline significantly in the absence of ovarian input and independently of GnRH. Expression of the genes encoding other members of the activin signaling pathway are unaffected by loss of ovarian hormonal feedback, highlighting their selective effect on *Foxl2*. Expression of *Gnrhr*, a known target of FOXL2, also declines upon ovariectomy consistent with reduced expression of *Foxl2* and loss of ovarian hormones. In contrast, *Fshb* mRNA increases dramatically post-ovariectomy due to increased compensatory input from GnRH. Together these data suggest that ovarian hormones regulate expression of *Foxl2* thereby expanding the number of genes controlled by the hypothalamic-pituitary-gonadal axis that ultimately dictate reproductive fitness.

## Introduction

Reproductive fitness requires tight regulation of the genes encoding LH and FSH. A complex network of signals emanate from the hypothalamic-pituitary-gonadal axis defining a characteristic temporal pattern of synthesis and secretion of LH and FSH that occurs over the course of the estrous cycle in rodents [1–3].

Peaks of LH and FSH that occur during late proestrus trigger ovulation [4]. This combined surge reflects primarily an increase in frequency and amplitude of pulses of GnRH from the hypothalamus [5–7]. Additional contributions from hypothalamic kisspeptin, pituitary adenylate cyclase-activating polypeptide, and pituitary bone morphogenetic proteins, as well as steroid feedback from the gonads, further define the frequency and amplitude of the preovulatory surge of LH and FSH [2, 8, 9].

A second surge of FSH occurs independently of LH during estrus. This secondary surge of FSH is less dependent on pulses of GnRH, which are slower and lower in amplitude during estrus, and instead reflects autocrine/paracrine increases in activin from pituitary gonadotropes and folliculostellate cells accompanied by reciprocal paracrine and endocrine decreases in follistatin from gonadotropes and folliculostellate cells, and inhibin from ovarian granulosa cells [2, 10–12]. The secondary surge of FSH signals the next round of follicular recruitment [10].

Growing evidence indicates that the activin signaling pathway may be the primary trigger that drives the secondary surge of FSH [2, 10–12]. As a member of the TGFβ superfamily, activin acts by binding to serine/threonine receptor kinases located on the plasma membrane of gonadotropes [3, 12, 13]. Activation of receptor kinases leads to phosphorylation of SMAD2/3, which releases from the receptor, associates with SMAD4, and translocates to the nucleus where the complex regulates transcription by binding to SMAD responsive elements [14]. While activin has been reported to regulate expression of both *Lhb* and *Fshb* [2], conditional, gonadotrope-specific knockout of SMAD4 led to a unique FSH deficient phenotype with normal levels of serum LH and *Lhb* mRNA [15]. This result is consistent with the notion that rising levels of activin during estrus may selectively regulate expression of *Fshb* that underlies the secondary surge of FSH.

The proximal promoter-regulatory region of the *Fshb* promoter contains conserved and species-specific regulatory elements that bind complexes of SMADs and the forkhead transcription factor FOXL2 [16–22]. These complexes form in response to activation of the activin signaling pathway and *in vitro* render the *Fshb* promoter responsive to the members of the TGFβ superfamily [2, 23–25]. Conditional, gonadotrope specific deletion of *Foxl2* in mice impairs fertility in both male and female mice due to FSH deficiency caused by diminished transcription of *Fshb* mRNA [23]. The importance of interactions between SMAD4 and FOXL2 is further underscored upon conditional, gonadotrope specific deletion of both genes in mice. While males remain fertile, females are sterile due to complete absence of FSH and undetectable expression of *Fshb* mRNA [15].

Expression of *Foxl2* is restricted to developing eyelids, ovarian granulosa cells, and pituitary thyrotropes and gonadotropes [26, 27]. Emerging evidence indicates that expression of *Foxl2* may be controlled by sex steroids. For example, estrogen up regulates expression of *Foxl2* in Southern catfish [28], rainbow trout [29], and the rare minnow, *Gobiocyprus rarus* [30]. In bovine endometrium, progesterone treatment decreases FOXL2 levels and its levels during the estrous cycle are inversely related to circulating progesterone levels [31]. Together, these studies suggest that the expression of *Foxl2* in gonadotropes may be subject to regulation by the hypothalamic-pituitary-gonadal axis (HPG).

Examining gonadotrope gene expression *in vivo* is challenging given the comingled arrangement of at least five cell types in the pituitary. Gonadotropes make up 5–15% of the cells in the pituitary [32, 33]. Roughly 80% of gonadotropes express both LH and FSH and after castration the number of cells co-expressing both hormones increases to close to 100% [34]. Other specialized cell types include somatotropes, corticotropes, lactotropes, thyrotropes and folliculostellate cells. This cellular complexity complicates study of cell-restricted or ubiquitous transcripts that differentially regulate the glycoprotein hormone encoding genes of the gonadotrope. To circumvent this limitation, we established a new line of mice (Gonadotrope specific RiboTag or GRT) by crossing mice that express CRE recombinase specifically in gonadotropes (*LHB-cre*) [35] with RiboTag mice [36] thereby allowing gonadotrope specific transcripts to be isolated by immunoprecipitation.

Herein we report the use of GRT mice, in conjunction with an ovariectomy (OVX)/GnRH antagonist paradigm, to interrogate the impact of loss of ovarian function on a battery of genes that define the activin signaling pathway. Our *in vivo* data suggest that ovarian hormones regulate expression of gonadotrope *Foxl2* thereby expanding the number of genes controlled by the HPG axis that ultimately dictate reproductive fitness.

## Materials and Methods

### Ethics Statement

All animal procedures were approved and carried out with strict accordance to guidelines from the Institutional Animal Care and Use Committee (IACUC, no. 4031) at Washington State University. Surgeries were performed under avertin anesthesia. Ketoprofen was used as the post-operative analgesic and all efforts were made to minimize animal suffering. Animals were euthanized using CO_2_.

### Animals

RiboTag mice (*Rpl22* ^*HA/HA*^) mice were generously provided by Dr. Paul S. Amieux (University of Washington) and have been described previously [36]. RiboTag mice harbor a floxed allele of *Rpl22* that encodes a component of the 60S ribosomal subunit. This allele was engineered to express RPL22 with a hemagglutinin (HA) tag upon CRE-mediated recombination. *LhbCre* (*Tg(Lhb-cre)1Sac*) mice were kindly provided by Dr. Sally Camper (University of Michigan) and have been previously described [35]. *Tg(Lhb-cre)1Sac* mice express Cre recombinase in gonadotropes due to *Lhb* promoter driven expression of Cre recombinase. This was confirmed by crossing *Tg(Lhb-cre)1Sac* mice with either a *RosaGFP* cre-reporter line or the *Rosa26LacZ* cre-reporter line. Both reporter genes were expressed primarily in gonadotropes and minimally in the testes of male mice along with a few cells in the cortex of the brain and the kidney [35]. This minimal expression in other tissues is nugatory due to the use of only the pituitary in the immunoprecipitations. *Rpl22* ^*HA/HA*^ mice were mated with *Tg(Lhb-cre)1Sac* mice to produce GRT mice.

Genotyping was performed on tail sections that were digested overnight in 10mM NaOH and 0.2mM EDTA at 70°C then PCR was performed using this lysate and primers specific for either the tagged HA-RPL22 locus or LhbCre (primer sequences in [35, 36]). Offspring containing both the tagged RPL22HA allele as well as the *Lhbcre* transgene were used in the HA-RPL22 isolation experiments.

Ovariectomies were performed on 8 to 18 week old mice. Fifteen-21 age matched GRT females were split into sham + vehicle, OVX + vehicle, or OVX + antide groups. Starting on the day of surgeries, female mice were injected with the vehicle propylene glycol or 60 μg Antide in propylene glycol every other day for 10 days at which point pituitaries were harvested and pooled from 5 to 7 animals based on treatment group with each group containing the same number of pituitaries within a given experiment. Pooled samples were divided into aliquots for analysis of total versus immunopreciptated RNA by real time PCR. Each point on the graph represents a pooled sample from a single experiment.

### Polyribosome immunoprecipitation

Immunoprecipitations were performed as described in Sanz *et al*. [36] with minor modifications. All steps were performed on ice. Briefly, magnetic Protein G beads (Dynabeads Protein G; Invitrogen) were conjugated to HA antibody (HA.11 Clone 16B12 monoclonal antibody; Covance) or Myc antibody (9E10; Santa Cruz) by rotation for one hour at room temperature. Beads were washed once in citrate buffer, twice in immunoprecipitation buffer, and then incubated with soluble pituitary extracts, as described below. Pituitaries were removed, pooled by treatment group (5–7 pituitaries per group), homogenized in ice-cold polysome buffer (400μL) [36], and centrifuged at 10,000g for 10 min. An aliquot of supernatant (input) was reserved with the remaining supernatant added to the HA-conjugated beads and incubated by rotation overnight at 4°C. The next day, beads were washed three times in high salt buffer for 5 min. The input fraction (pituitary) and the immunoprecipitated fraction (gonadotrope specific) was treated with 5U DNase (Fermentas) by incubation at 25°C for 15 min and then 37°C for 15 min followed by incubation on ice. Qiagen RLT buffer was then added to the DNase-treated samples and RNA isolated using the RNeasy MinElute Cleanup protocol (Qiagen). All samples were stored at -80°C. RNA was analyzed using either a NanoDrop Spectrophotometer (Thermo Scientific) or a Eukaryote Total RNA Pico Chip on the Agilent 2100 Bioanalyzer (Washington State University Laboratory for Bioanalysis and Biotechnology I).

### cDNA synthesis and qPCR

cDNA was synthesized using qScript cDNA SuperMix (Quanta Biosciences) following the manufacturer’s protocol using the input fraction (total pituitary) or the immunoprecipitated fraction (gonadotrope) RNA. The synthesized cDNA was diluted 1:15, and 5 μL was used in each subsequent real time reaction using Fast SYBR Green Master Mix (Applied Biosystems) in a 20 μL final volume reaction on a 7500 Fast real time machine (Applied Biosystems). Cycling conditions were incubation at 95°C for 20 sec followed by 40 cycles of 95°C for 3 sec then 60°C for 30 sec followed by melt curve analysis. Primers were optimized for primer concentration, and primer efficiency was determined. Only primer sets with primer efficiencies between 95% and 105% were used. Primer sequences and concentrations are listed in Table 1. *Rpl19* was used as the endogenous control and cDNA from LβT2 cells was used as the calibrator in the 2^-ΔΔCt^ calculation [37]. qPCR for each primer set was performed in duplicate for each sample isolated and the ΔCt mean used in calculations. 2^-ΔCt^ or 2^-ΔΔCt^ values were graphed for experiments performed on different days with the median with interquartile range shown. Although infrequent, amplicon values with high Ct values that exceeded the detectable range as determined by standard curve analysis were excluded. Thus “n” values ranged from 3 to 5 for any given primer set.

**Table 1:** Transcripts, primer concentrations and primer sequences used in real time analysis.

Transcript	Concentration	5'→3'
*Lhb*	300 nM	CACCTTCACCACCAGCATCT
		GCACAGGAGGCAAAGCA
*Gnrhr*	300 nM	TTGTCTTTGCAGGACCACAGTTAT
		TGGGTCACACATTGCGAGAA
*Cga*	150 nM	TGCTTCTCCAGGGCATATCC
		CTTTGGAACCAGCATTGTCTTCT
*Fshb*	300 nM	TGCCGTTTCTGCATAAGCAT
		TCGTATACCAGCTCCTTGAAGGTA
*Rpl19*	150 nM	GGAAAAAGAAGGTCTGGTTGGA
		TGATCTGCTGACGGGAGTTG
*Gh*	150 nM	CCCAGGCTGCTTTCTGCTT
		AGCAATTCCATGTCGGTTCTCT
*Prl*	300 nM	CCCTGGCTACACCTGAAGACA
		GACTGCACCAAACTGAGGATCA
*Tshb*	300 nM	CACCATCTGTGCTGGGTATTGT
		GTGCATATTTGGGAAGAAACAGTTT
*Foxl2*	150 nM	TCCGGCATCTACCAGTACATCA
		TATTCTGCCAGCCCTTCTTGTT
*Inhba*	150 nM	AGAAGGCAACCACACGACTTTT
		CTTGCCAACAGAAATCCTCTCA
*Inhbb*	300 nM	AGACATCGCATCCGCAAAC
		GATGAAGAACTGTTGCCTGCAA
*Inha*	300 nM	TGCACAGGACCTCTGAACCA
		GGCACCTGTAGCTGGGAAAA
*Smad2*	300 nM	TTGTGCAGAGCCCCAACTG
		CGGCTTCAAAACCCTGGTT
*Smad3*	300 nM	CTCTCCCCGAATCCGATGT
		AGGCCGGCTCACAGTAGGT
*Smad4*	150 nM	AGACTACCCCAGGCAGAGCAT
		GCTCGGTGAAGGTGAATCTCA
*Smad7*	300 nM	CCATCAAGGCTTTTGACTATGAGA
		AAGCTGATCTGCACGGTGAA

### Statistical Analysis

Data from the separate experiments were combined and analyzed with the assumption that there was no significant interaction between the “experiment” and treatment group and no “effect of experiment.” While this approach introduces additional independent variables that can increase biological variability, it allows for a more conservative estimate of statistical significance.

Statistical analysis was performed using one-way parametric ANOVA followed by Tukey’s multiple comparison test (*<0.05; **<0.01; ***<0.001) with GraphPad software.

## Results

### Gonadotrope specific transcripts are enriched in polyribosomes isolated from GRT mice

**Fig 1:**
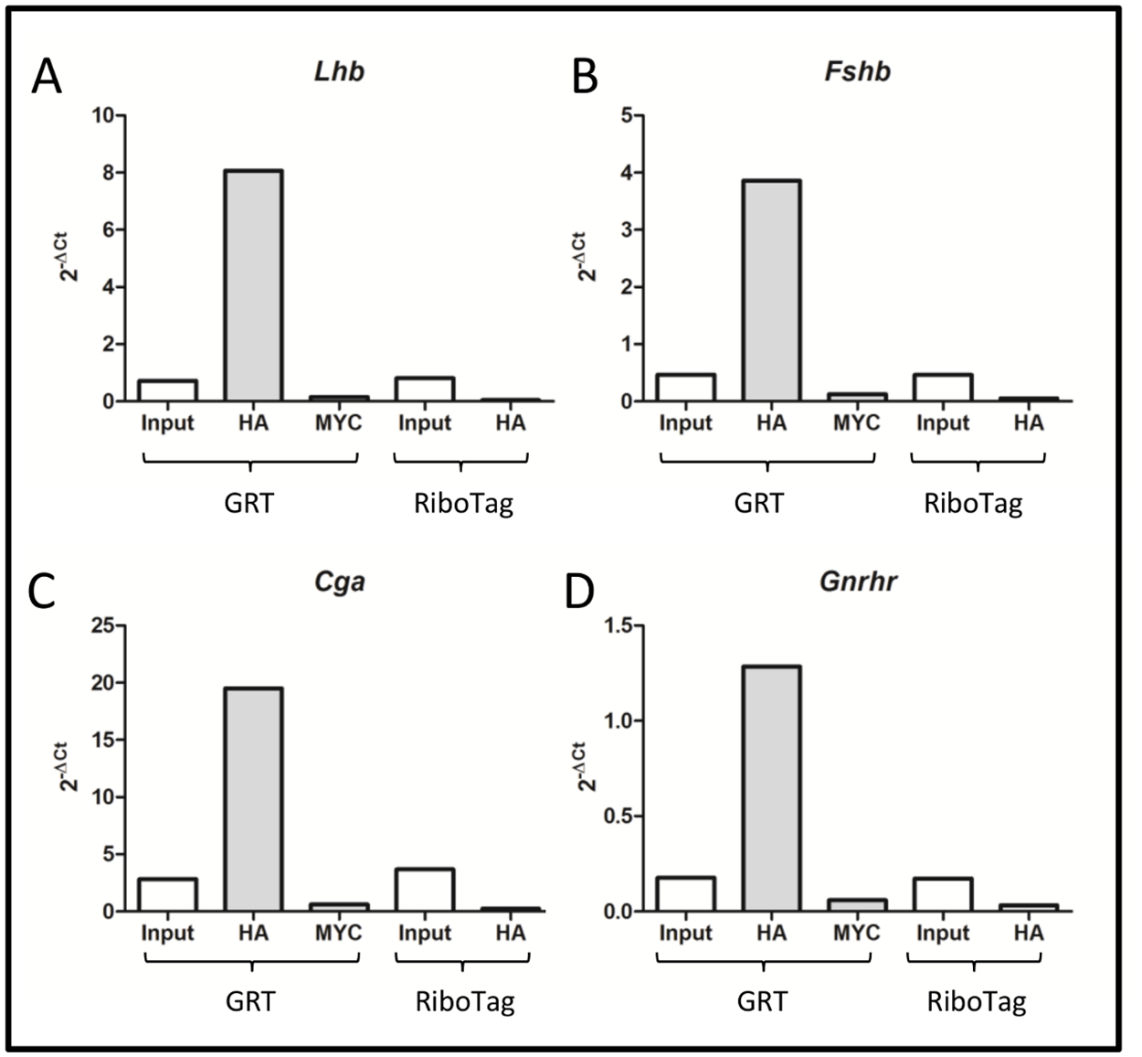
Gonadotrope specific transcripts are enriched in polyribosomes isolated from GRT mice. Pituitaries were isolated from five GRT or RiboTag female mice and pooled. Isolated RNA was subject to immunoprecipitation with either HA or Myc antibody. Real-time PCR was performed with primers specific to A.) *Lhb*; B.) *Fshb*; C.) *Cga*; and D.) *Gnrhr*. *Rpl19* primers were used for normalization in the calculation of 2^-ΔCt^ values.

Gonadotrope specific RiboTag (GRT) mice express HA-tagged RPL22 protein exclusively in gonadotropes. To verify isolation of gonadotrope specific transcripts, pituitaries were harvested from GRT mice as well as RiboTag mice, which lack the *Lhbcre* transgene and serve as a control. Lysates were subject to immunoprecipitation with either HA or Myc antibody. Gonadotrope specific transcripts (*Lhb*, *Fshb*, *Cga*, and *Gnrhr*) were enriched (11-fold, 8-fold, 7-fold, and 7-fold, respectively) in HA pulldowns from GRT pituitary lysate (Fig 1). The enrichment is specific to the HA tag present on the RPL22 protein as neither immunoprecipitation with Myc antibody from pituitaries of GRT mice nor immunoprecipitation with HA antibody from control Ribotag mice showed enrichment over the input values (Fig 1).

### GnRH responsive changes in gonadotrope specific mRNAs from HA-tagged polyribosomes mirror that observed in total pituitary RNA

Levels of *Lhb*, *Fshb*, and *Cga* mRNAs in murine pituitaries increase after castration [38] while *Gnrhr* mRNA levels decrease [39, 40]. Treatment with the GnRH antagonist Antide inhibits the post-castration rise of *Lhb*, *Fshb*, and *Cga* mRNA [41, 42], providing a measure of GnRH responsiveness. To determine whether the amount of polyribosomal associated transcripts follow these patterns, GRT mice were either sham operated or OVX followed by a 10-day treatment with either vehicle or Antide. Pituitaries were then harvested and transcripts isolated from both total pituitary RNA (input fraction) and HA-tagged polyribosomes (gonadotrope specific fraction).

**Fig 2:**
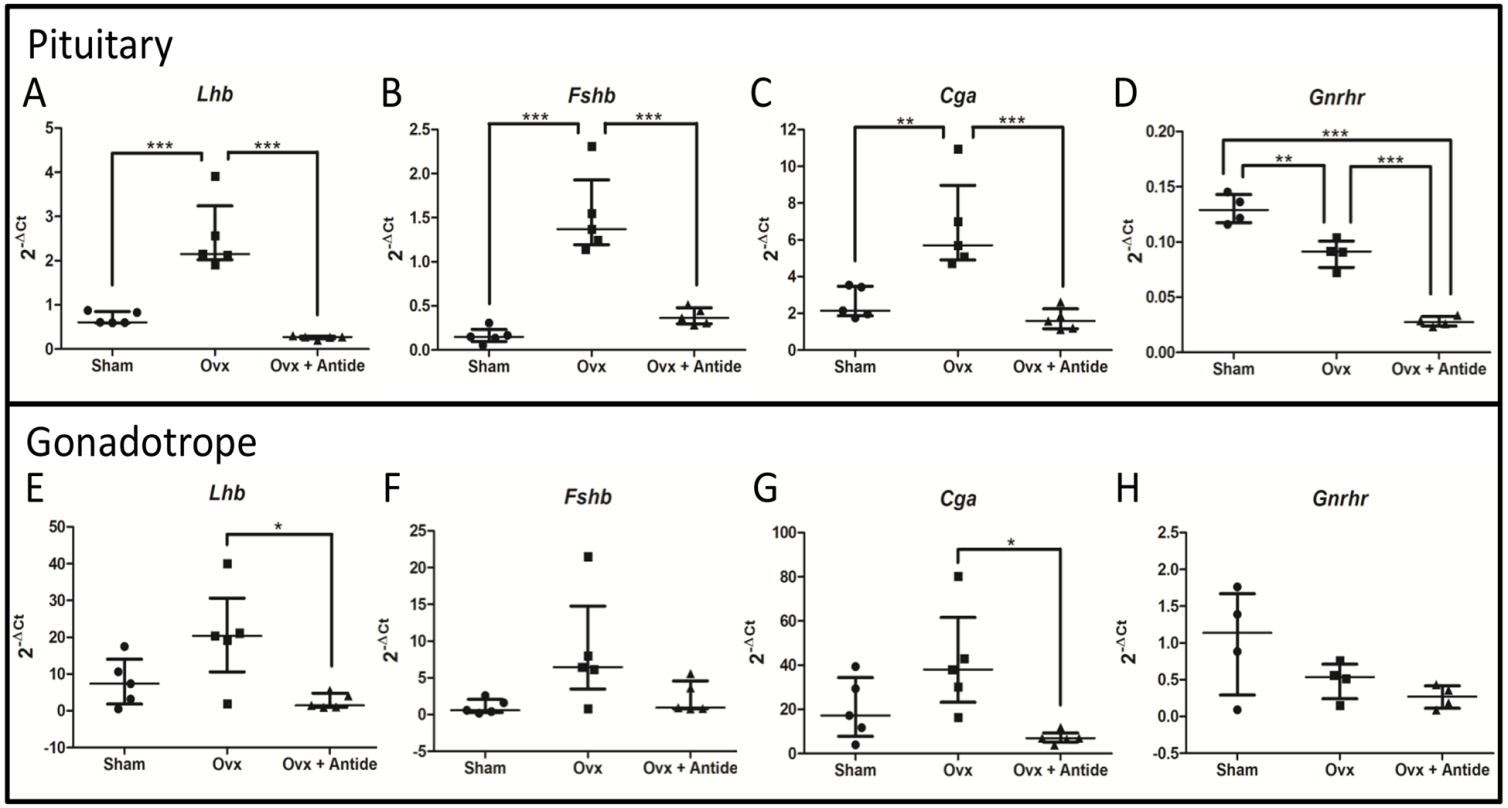
GnRH responsive transcripts increase after ovariectomy and decrease after Antide treatment. Gonadotrope specific transcripts were isolated from female mice either sham operated or OVX and then treated with either vehicle or Antide. Real-time PCR 2^-ΔCt^ values for five separate experiments (n = 5) were plotted showing the median with interquartile range of total pituitary levels of A.) *Lhb*; B.) *Fshb*; C.) *Cga*; and D.) *Gnrhr* and gonadotrope levels of E.) *Lhb*; F.) *Fshb*; G.) *Cga*; and H.) *Gnrhr*. Statistical differences were determined by a one-way ANOVA followed by Tukey’s multiple comparison test (*<0.05; **<0.01; ***<0.001). Each “n” represents 5–7 pooled pituitaries per treatment group.

Changes in the levels of three gonadotrope-specific transcripts (*Lhb*, *Fshb*, and *Cga*) in total pituitary RNA mirrored changes of the three mRNAs in HA-tagged polyribosomal mRNA from gonadotropes (Fig 2; panels A-C compared with panels E-G). Gonadotrope levels showed more variance than total pituitary levels, possibly a result of differences in ribosome loading between samples or other technical differences associated with immunoprecipitation of small sample sizes. This variance prevented demonstration of statistical significance for some of the treatment groups. Nevertheless, the trends mirror those seen for total pituitary mRNA.

As expected, mRNAs for *Lhb*, *Fshb*, and *Cga* increased after OVX, reflecting removal of steroid negative feedback that should be accompanied by a subsequent increase in GnRH secretion. Respective mRNA fold-changes (total versus polyribosomal) were 3.6- vs. 2.6-fold for *Lhb*, 9.4- vs. 8.0-fold for *Fshb*, and 2.6 vs. 2.0-fold for *Cga*. Similar mirrored changes in levels of *Lhb*, *Fshb*, *Cga* mRNAs from OVX + Antide treated mice were also observed when measured in either total RNA or in polyribosomes from gonadotropes. Indeed, Antide almost completely blocks the post-OVX rise in *Lhb*, *Fshb*, and *Cga*, which is expected if their increase is dependent primarily on GnRH.

In contrast to *Lhb*, *Fshb*, or *Cga* mRNA, *Gnrhr* mRNA exhibited a post-OVX decline that was similar when measured either in total pituitary RNA or in polyribosomes from gonadotropes (approximately 70% vs. 50%, respectively). Treatment of OVX animals with Antide led to an even further significant decrease in *Gnrhr* mRNA (30%) compared to OVX alone in samples of total pituitary RNA.

**Fig 3:**
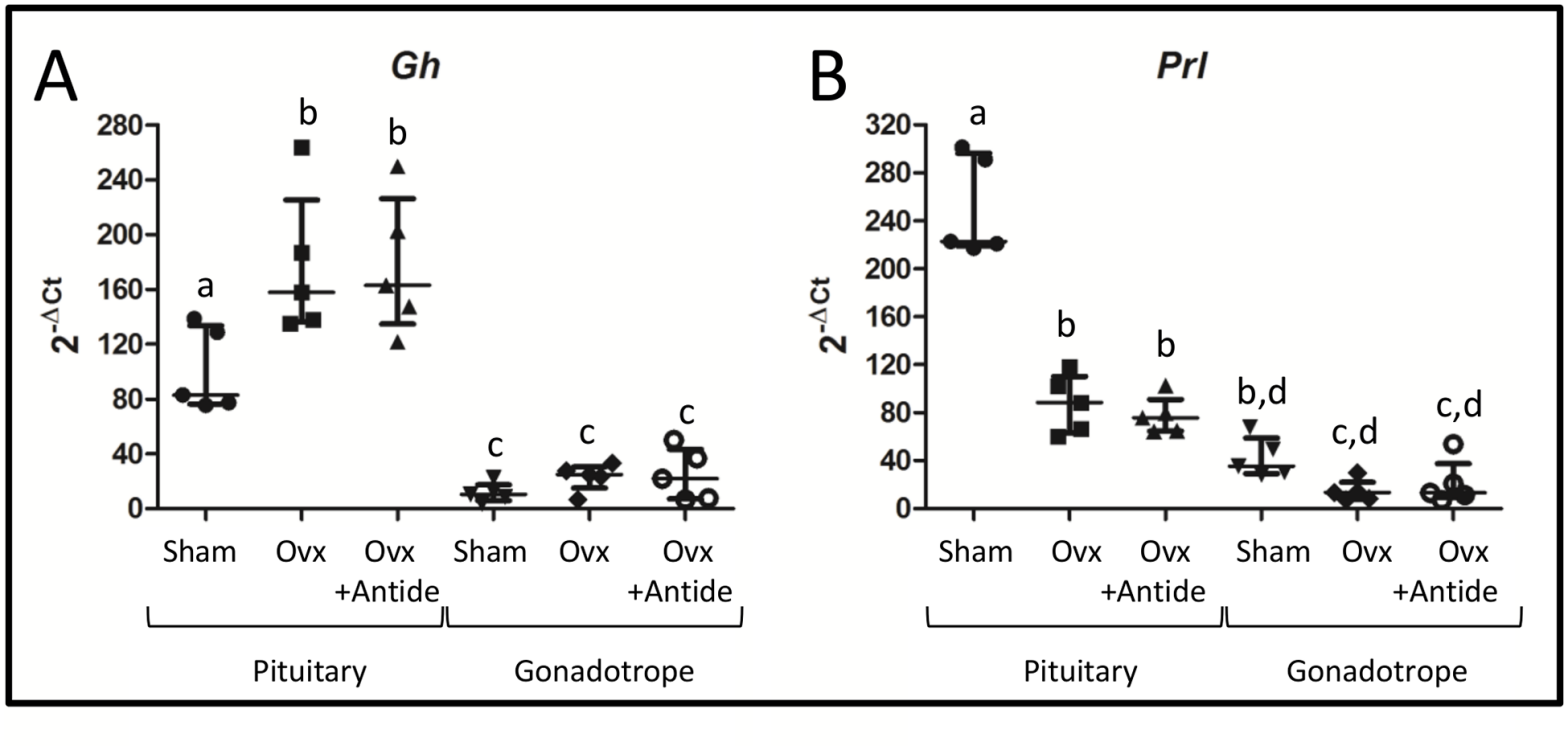
*Gh* and *Prl* mRNAs lack enrichment in HA-tagged polyribosomes. Real-time PCR was performed on total RNA or HA-tagged polyribosomes with primers specific to A.) *Gh* and B.) *Prl*. Values were plotted with the median and interquartile range values indicated for both total pituitary and HA-tagged polyribosomes. Statistical differences (n = 5) were determined by a one-way ANOVA followed by a Tukey’s multiple comparison test. Groups identified with different letters indicate significantly different groups. Each “n” represents 5–7 pooled pituitaries per treatment group.

To further verify that HA-tagged polyribosomes are enriched with transcripts from gonadotropes, levels of the somatotrope specific *Gh* mRNA and the lactotrope specific *Prl* mRNA were also examined. *Gh* mRNA exhibits a post-OVX rise whereas *Prl* mRNA declines (Fig 3). This result is consistent with the known negative and positive effects of gonadal steroids on *Gh* and *Prl* mRNAs, respectively [43, 44]. Moreover, both of these non-gonadotrope transcripts were significantly depleted in HA-tagged polyribosomes when compared to their levels in total pituitary RNA (Fig 3). This result is expected if the polyribosomes are derived primarily from gonadotropes. Finally, levels of both mRNAs were refractory to treatment with Antide, which is expected since GnRH receptors are uniquely located in the pituitary on gonadotropes [45].

### Activin and inhibin subunit transcript levels in gonadotropes are refractory to regulation by ovarian hormones or GnRH

*Fshb* expression is induced by activin [46]. Activin A and activin B are homodimers of the inhibin βA (Inhba) or inhibin βB (INHBB) subunits, respectively. Inhibin, an antagonist to activin signaling, is also present in two forms, inhibin A and inhibin B, heterodimers of a common α subunit (INHA) and either INHBA or INHBB subunits, respectively. These factors act as autocrine and paracrine signals in the pituitary [47–49]. Given that activin and inhibin are widely expressed in a variety of tissues including ovary, brain, placenta, kidney, adrenal, and bone marrow [47], GRT mice should be useful for measuring changes in their subunit mRNAs in the gonadotrope using the OVX/Antide paradigm as different responses to OVX have been reported [38, 40].

**Fig 4:**
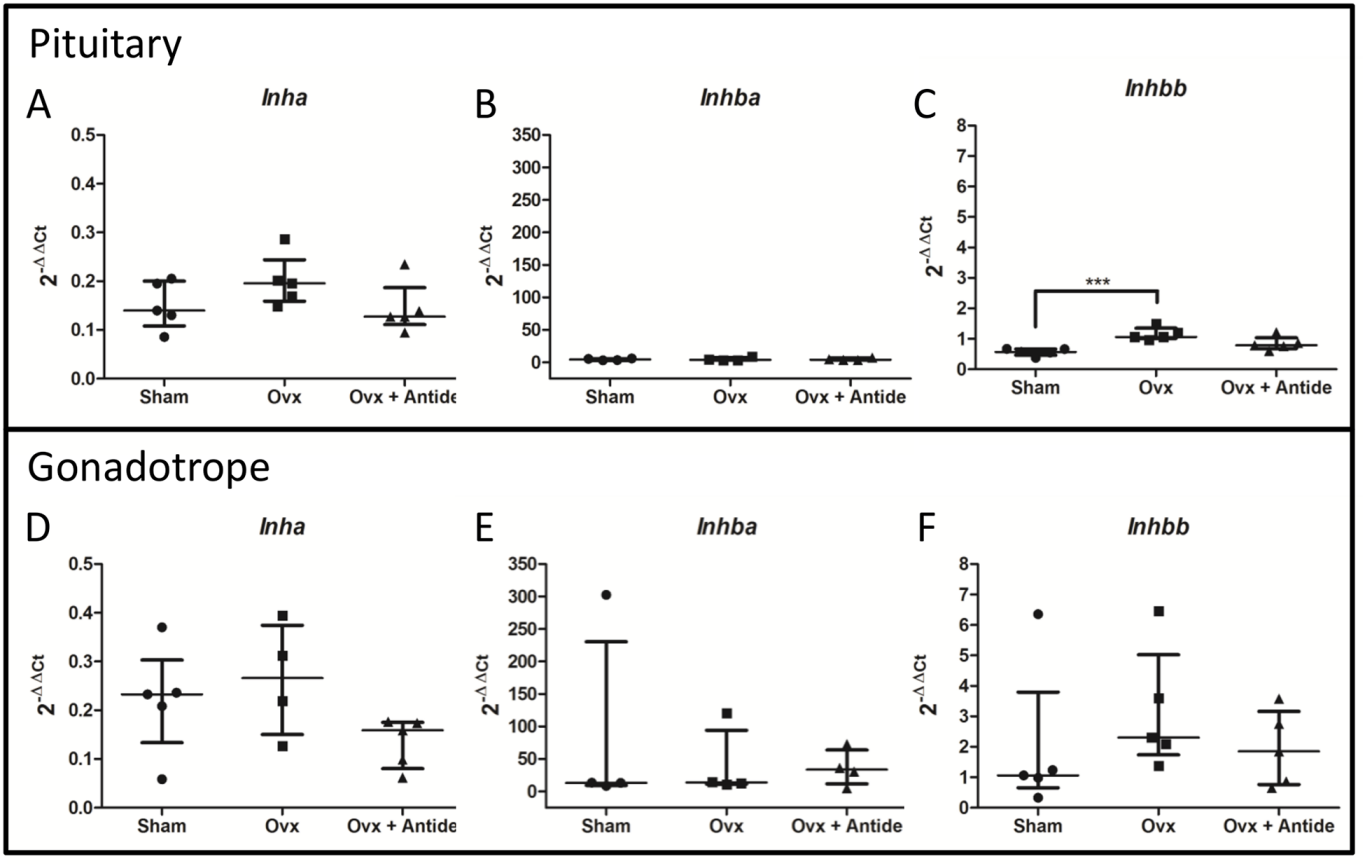
Activin and inhibin subunit transcript levels are refractory to regulation by ovarian hormones or GnRH. 2^-ΔΔCt^ values were calculated using total RNA from LβT2 cells as the calibrator. The median with the interquartile range is indicated for total pituitary levels of A.) *Inha*; B.) *Inhba*; and C.) *Inhbb* and gonadotrope levels of D.) *Inha*; E.) *Inhba*; and F.) *Inhbb*. Statistical differences (n = 5) were determined by a one-way ANOVA followed by Tukey’s multiple comparison test (***<0.001). Each “n” represents 5–7 pooled pituitaries per treatment group.

Results from the GRT mice indicate that OVX has a minimal impact on the levels of activin and inhibin subunit mRNAs in total pituitary extracts, with the exception of *Inhbb* mRNA increasing by two-fold after OVX (Fig 4A, 4B and 4C). Similarly in gonadotropes, levels of these subunit mRNAs appear to be independent of ovarian and GnRH input, although levels are more variable (Fig 4D, 4E and 4F). While comparison of the 2^-ΔΔCt^ values suggests that *Inhbb* mRNA may be enriched by approximately two-fold in gonadotropes, analysis by the Wilcoxon-Mann-Whitney test indicates that the change is suggestive but inconclusive statistically (p = 0.095). Nevertheless, the putative enrichment of *Inhbb* mRNA is consistent with previous reports indicating that activin B is expressed at higher levels in gonadotropes compared to other cells of the pituitary [49].

Together, our results suggest that even though activin and inhibin are important regulators of *Fshb* expression [2, 3, 12], their subunit mRNA levels in the gonadotrope appear to be independent of the ovary and refractory to the changes in GnRH that occur with the OVX/Antide paradigm.

Attempts were made to measure the level of follistatin, another target of *Foxl2* and *Smad3* [25] and itself a regulator of activin signaling, yet levels were too low in these samples to be readily detectable (data not shown) as also has been found by others [38].

### *Smad3* mRNA in gonadotropes may be ovarian dependent and GnRH independent

Activin signals through SMAD proteins and *Smad3*-deficient male mice have lower levels of *Lhb* and *Fshb* [50]. AP1 and SMAD proteins synergize to regulate *Fshb* [51], offering evidence of the interplay between GnRH and activin signaling in the control of *Fshb* expression. Like activin and inhibin, SMADs are widely expressed in cells and tissues, thus the GRT mice allow for assessment of effects of the ovarian hormones and GnRH on *Smad* mRNAs specifically in gonadotropes.

**Fig 5:**
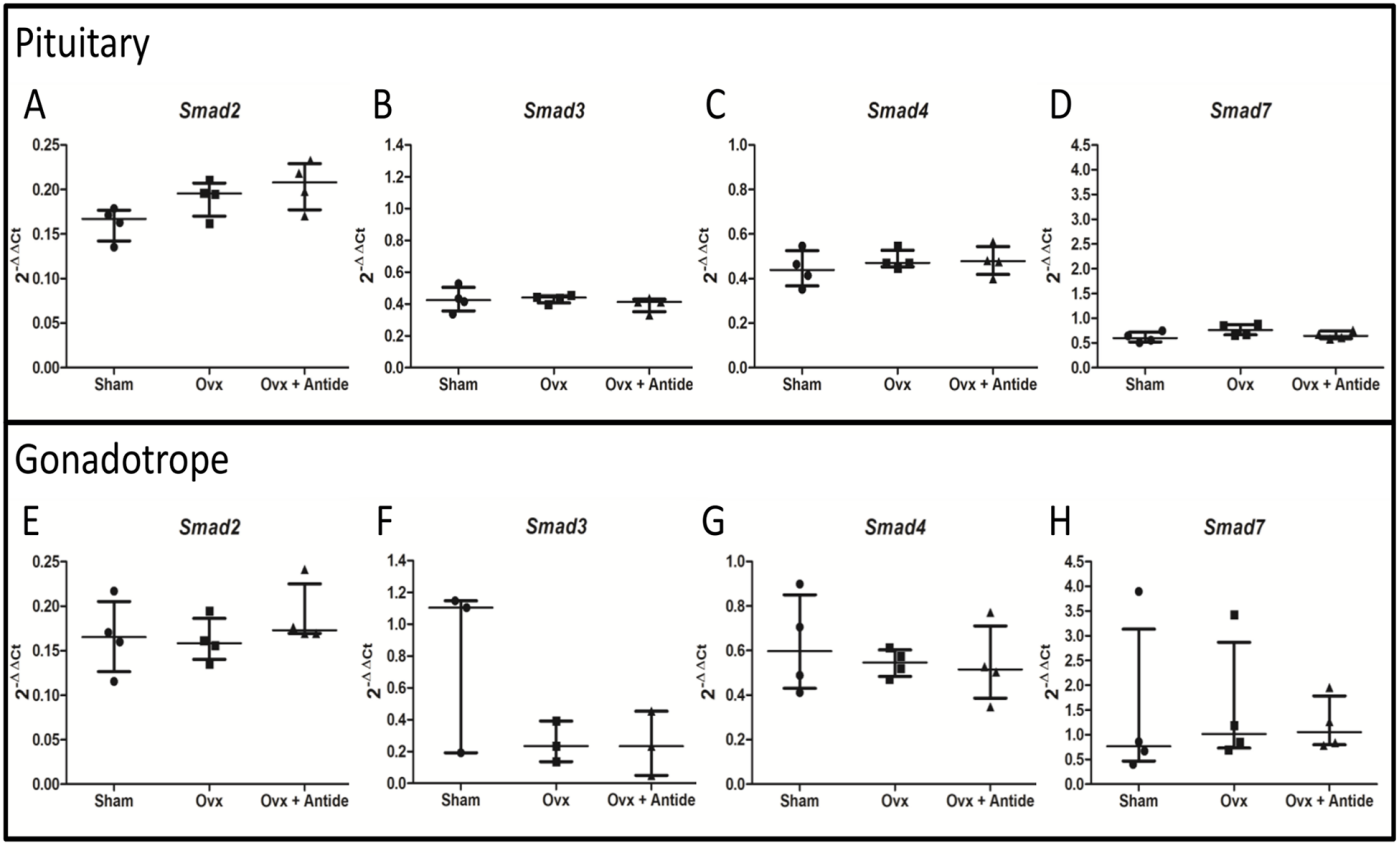
*Smad3* mRNA in gonadotropes may be ovarian dependent and GnRH independent. 2^-ΔΔCt^ values were calculated using total RNA from LβT2 cells as the calibrator and these levels were plotted with the median indicated along with the interquartile range for pituitary A.) *Smad2*; B.) *Smad3*; C.) *Smad4*; and D.) *Smad7* and the gonadotrope E.) *Smad2*; F.) *Smad3*; G.) *Smad4*; and H.) *Smad7*. Statistical differences were determined by a one-way ANOVA followed by Tukey’s multiple comparison test. Here n = 3 or 4, where “n” represents 5–7 pooled pituitaries per treatment group).

Removal of the ovary with or without Antide treatment has little impact on *Smad3*, *Smad4*, or *Smad7* when sampled from total pituitary RNA (Fig 5B, 5C and 5D). Although, *Smad2* mRNA levels trend higher after OVX with levels that remain unaffected after treatment with Antide (Fig 5A), the change lacks statistical significance. Changes in *Smad* mRNAs in gonadotropes also lacked statistical significance, although *Smad3* mRNA may be dependent on the ovary and independent of GnRH given that one outlier prevented demonstration of statistical significance. The observation that *Smad3* mRNA is enriched in the gonadotrope fraction when compared to total pituitary RNA (2^-ΔΔCt^ values differ by a factor of two) further supports the notion that SMAD3 may have a greater role in gonadotropes than in other pituitary cell types.

### *Foxl2* levels in gonadotropes are dependent on the ovary

Both gonadotropes and thyrotropes synthesize FOXL2 as evidenced by its immunohistochemical co-localization with both LHβ and TSHβ [25, 52]. Hence, the GRT mouse provides a useful tool for assessing the impact of ovarian hormones and GnRH on the presence of *Foxl2* in polyribosomes from gonadotropes.

**Fig 6:**
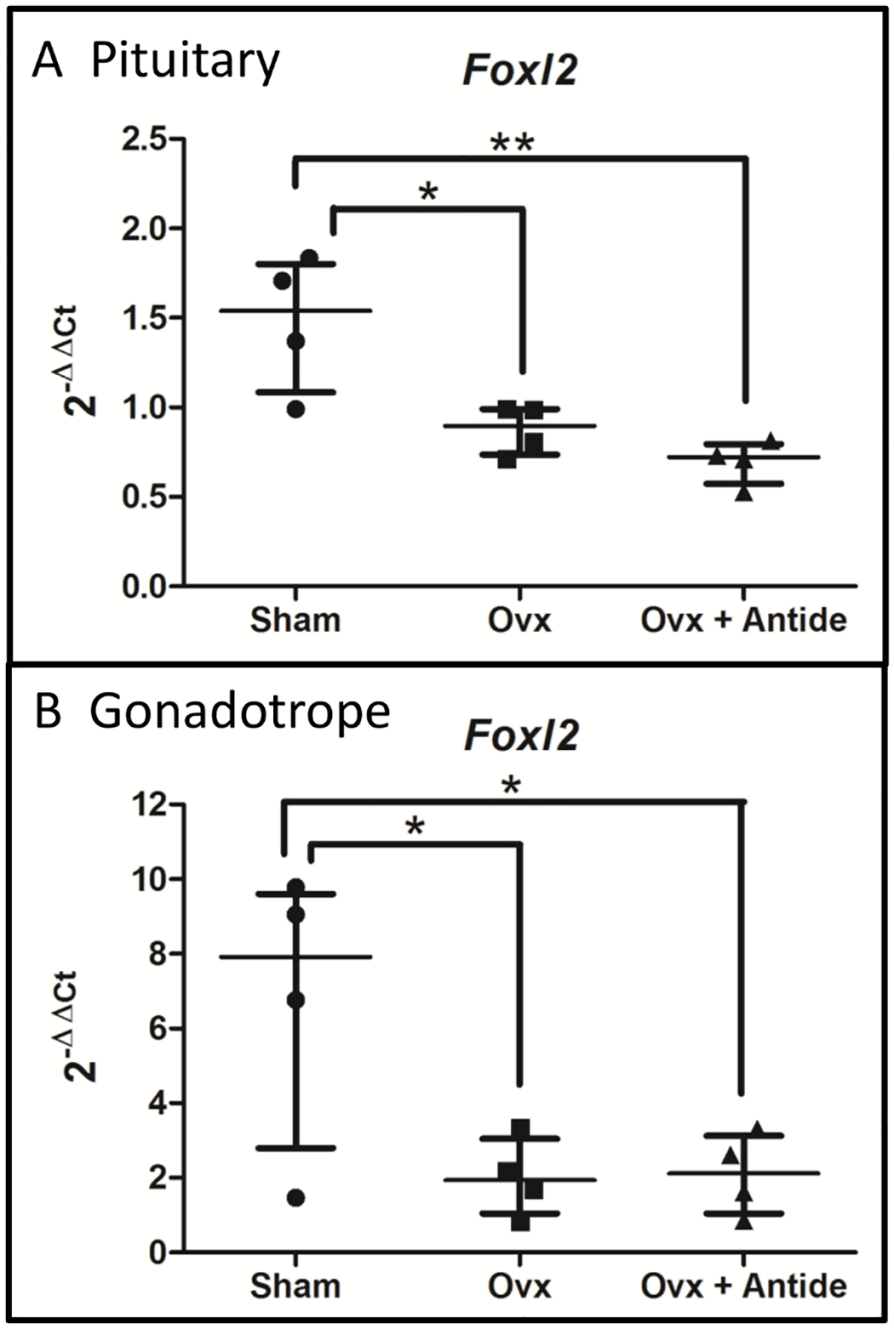
*Foxl2* levels in gonadotropes are dependent on the ovary. 2^-ΔΔCt^ values were calculated using total RNA from LβT2 cells as the calibrator. The median with interquartile range is shown for A.) total pituitary *Foxl2* levels and B.) gonadotrope *Foxl2* levels. Statistical differences were determined by a one-way ANOVA followed by Tukey’s multiple comparison test (*<0.05; **<0.01). N = 4, where each “n” represents 5–7 pooled pituitaries per treatment group.

*Foxl2* mRNA levels were enriched approximately five-fold in polyribosomal mRNA samples prepared from gonadotropes versus total pituitary RNA (Fig 6A and 6B). Lack of thyrotrope transcripts was confirmed by the absence of *Tshb* transcripts in the HA antibody pull-down (data not shown). Ovariectomy reduced *Foxl2* mRNA ~42% in total RNA samples and ~76% in polyribosomal RNA from gonadotropes. This differential suggests that the decrease in *Foxl2* mRNA occurs to a greater degree in gonadotropes than in thyrotropes. Antide had no impact on *Foxl2* levels in either total pituitary RNA or total gonadotrope polyribosomal RNA, suggesting that ovarian hormones play an important role in maintenance of *Foxl2* mRNA in gonadotropes. Interestingly, this decrease in *Foxl2* levels after OVX appears to be specific to females as *Foxl2* mRNA levels in intact males are not significantly different from levels in castrated males treated with vehicle or Antide (S1 Fig).

## Discussion

FOXL2, in partnership with SMAD4, has been characterized recently as an essential master regulator of fertility via its positive effects on *Fshb* gene expression *in vivo* [15]. Expression of *Fshb* in late embryonic gonadotropes also requires FOXL2 [22]. *In vitro* studies indicate that FOXL2 appears to act as a key transcriptional bridge that underlies the synergistic response of *Fshb* to GnRH, activin, glucocorticoids, and progestins [2, 22, 24, 53]. Together these studies offer compelling evidence supporting a central role for FOXL2 in regulating expression of *Fshb* in cycling females. Our current study suggests that levels of *Foxl2* mRNA in gonadotropes are regulated by ovarian hormones, thereby placing this transcriptional factor under control of the HPG axis. In this regard, *Foxl2* joins the immediate early genes (including *Egr1*, *Jun*, and *Fos*) as transcriptional targets of the HPG axis that play key roles in regulating gonadotrope gene expression.

### Increased GnRH compensates for diminished expression of *Foxl2* after ovariectomy

Expression of *Fshb* in gonadotropes increases by approximately eight-fold after OVX (Fig 2). Treatment with Antide almost completely blocks the post-OVX increase in *Fshb* mRNA. This result is consistent with the known increase in GnRH pulse frequency that occurs after OVX that is due to a loss of negative feedback from gonadal steroids [54, 55]. Although removal of the ovary also reduces serum inhibin, its loss appears to contribute only marginally to the increase in *Fshb* mRNA given the almost complete block of the post-OVX rise that occurs after Antide treatment.

Activin B has been linked with the rise in FSH secretion after OVX in the rat by use of passive immunoneutralization with monoclonal antibody generated against activin B [56, 57]. Nevertheless, our results suggest that activins produced in gonadotropes are unaffected by OVX as no significant changes occurred in the levels of *Inha* and *Inhba* mRNAs (Fig 4D and 4E). Even though OVX led to a modest increase in *Inhbb* mRNA, the robust reduction in *Foxl2* mRNAs in gonadotropes makes it unlikely that changes in activin signaling account for the substantial increase in *Fshb* mRNA.

Although activin can act in an autocrine manner to induce *Fshb* in the pituitary [9, 57, 58], other tissue sources of activins and inhibin could also contribute to their serum levels. Yet, despite these possible sources of activins, the mRNA for the downstream signaling factor FOXL2 decreased after OVX and was refractory to changes in GnRH (Fig 6). In fact, *Foxl2* expression decreased by almost 50% in total pituitary RNA and by an additional 50% in polyribosomal RNA from gonadotropes. Together, these changes suggest that activin signaling may be less important than GnRH in mediating the post-OVX rise in expression of *Fshb*. This notion is also consistent with previous findings indicating that the long-term post OVX rise in *Fshb* is GnRH dependent [59].

### Maintenance of *Foxl2* and *Gnrhr* expression requires ovarian hormones

While it is well known that loss of steroid negative feedback increases synthesis and secretion of GnRH [60], the impact of ovarian hormones on *Foxl2* expression is less understood. In that regard, we were surprised that OVX led to substantial decreased expression of *Foxl2* and that the decline occurred independently of GnRH (Fig 6). The dependence of *Foxl2* expression on ovarian hormones suggests that their role may be to ensure that *Foxl2* transcripts are sufficiently abundant during late proestrus to support the secondary surge of FSH secretion that occurs during estrus, a period when expression of *Fshb* is less dependent on GnRH and more dependent on activins.

To date, little is known about the regulation of *Foxl2* transcription. The *Foxl2* promoter is bidirectional [61] with the LIM homeodomain factors *Lhx3* and *Lhx4* [52], as well as *Foxl2* itself, [62, 63] up-regulating its transcription. Nicotinamide, a SIRT1 inhibitor, has been shown to increase *Foxl2* transcript and protein levels in granulosa cells [63]. In our OVX/Antide treatment paradigm, *Foxl2* regulation appears to be primarily the result of ovarian factors as blocking GnRH activity with Antide treatment did not relieve the repression seen after OVX. It is also interesting that in males, orchiectomy did not change the level of *Foxl2* mRNA (S1 Fig), further supporting the view that estrogens or progestins may be the ovarian hormones that maintain *Foxl2* expression during the estrous cycle.

Expression of *Gnrhr* mRNA, when measured in total RNA, also declined after OVX and fell even further after treatment with Antide, suggesting that levels of the receptor mRNA are dependent on both the ovary and GnRH (Fig 2D). In contrast, when measured in HA-tagged polyribosomes, *Gnrhr* mRNA levels appeared completely dependent on the ovary and independent of GnRH. This outcome may reflect selective isolation of *Gnrhr* mRNA from a subpopulation of gonadotropes that only express *Lhb* mRNA since the *LhbCre* transgene would be silent in cells that only express *Fshb*. This possibility is consistent with previous reports indicating that GNRHR levels decrease by 50% after OVX [64] and that treatment of *hpg* mice with estradiol and exogenous GnRH can restore GNRHR to normal levels [65]. Additionally, treatment of OVX mice with GnRH antiserum led to even further decline in GNRHR [39].

FOXL2 also regulates expression of *Gnrhr* in αT3 cells, a gonadotrope-specific cell line [27]. Thus, the post-OVX decline in *Gnrhr* could be due to down regulation of *Gnrhr* mRNA caused by an increase in GnRH, or loss of a positive contribution from gonadal steroids that regulate FOXL2, or by a combination of changes in both. We favor the latter possibility as treatment of OVX animals with Antide led to an even further significant decrease in *Gnrhr* mRNA (30%) in samples of total pituitary RNA. This result suggests that levels of *Gnrhr* mRNA are dependent on gonadal steroids in intact animals and that elevated levels of GnRH that occur after OVX may provide partial compensation rather than down regulate the receptor encoding mRNA. Finally, treatment of OVX mice with estrogen and progesterone partially rescued GNRHR levels even in the presence of GnRH antiserum, a result which is consistent with our evidence that *Gnrhr* expression is dependent on ovarian steroids [39].

### Regulated expression of *Foxl2* during the rodent estrous cycle may function as a synergistic rheostat

Several regulatory elements within the proximal promoter of *Fshb* mediate responsiveness to GnRH and activin [2, 24]. These bind a plethora of transcriptional factors including glucocorticoid receptor, progesterone receptor, AP1, SMAD3, SMAD4, and FOXL2. All of these transcription factors are known to mediate the synergistic interactions between GnRH, activin, gonadal and adrenal steroids that ultimately define the transcriptional tone of *Fshb* [2, 15, 24].

At least two regulatory elements bind FOXL2 [2, 15, 19, 50]. These FOXL2 elements lie adjacent to or overlap with SMAD responsive elements [22]. Importantly FOXL2 and SMADS interact synergistically upon binding to their cognate response elements on the *Fshb* promoter [19]. Although GnRH signals primarily through an AP1 response element in the proximal *Fshb* promoter, recent *in vitro* studies indicate that cJUN interacts with FOXL2, with both proteins required for GnRH-responsiveness [22, 27]. Together these findings define a central role for FOXL2 as it serves as a bridge that joins both the GnRH and activin pathways that act positively to regulate transcription of *Fshb* over the estrous cycle.

Given that maximal expression of *Foxl2* in gonadotropes requires ovarian hormones, we posit that the concentration of gonadotrope *Foxl2* mRNA changes during the course of the rodent estrous cycle. While both estradiol and progesterone have been reported to regulate *Foxl2* expression [28–31], we suspect that progesterone plays a predominate role in regulating *Foxl2* expression in gonadotropes. Since the secondary surge of FSH is more dependent on activin than GnRH [2, 10–12], we predict that gonadotrope FOXL2 reaches maximal levels during late proestrus, a time when progesterone peaks and estradiol falls. If true, then the synergistic effect of FOXL2 and SMADs on expression of *Fshb* would be maximal at the time activin signaling becomes the primary driver of the secondary surge of FSH. As progesterone falls during estrus, FOXL2 levels may decline as well. One of the hallmarks of a synergistic interaction is that a small change in concentration of one of the interacting components can lead to a large biological effect. Thus, even modest changes in levels of *Foxl2* expression and its cognate protein would be expected to have an impact on expression of *Fshb* given the known synergism between FOXL2 and members of the SMAD family of DNA-binding proteins [19]. Progesterone receptor also interacts synergistically with the GnRH and activin signaling pathways. Thus, changes in progesterone levels may act as a rheostat that provides a one-two punch that mediates fluctuations in expression of *Foxl2* and activity of the progesterone receptor, both of which set the transcriptional tone of *Fshb* and ultimately fertility in females. Clearly future studies are needed to address the validity of this proposed model. Regardless of whether the model is correct, our collective data indicate that the HPG axis controls expression of *Foxl2* in pituitary gonadotropes primarily through positive feedback from ovarian hormones.

## Supporting Information

S1 Fig *Foxl2* levels in gonadotropes are independent of the testes. Male mice were either sham operated or orchiectomized and then treated with vehicle or Antide every other day for 10 days. Pituitaries were harvested and gonadotrope specific transcripts isolated. Real time PCR was performed with primers specific to *Foxl2* and *Rpl19*, used for normalization. 2^-ΔΔCt^ values were calculated using total RNA from LβT2 cells as the calibrator. The median with interquartile range is shown for A.) total pituitary *Foxl2* levels and B.) gonadotrope *Foxl2* levels. Statistical analysis was performed with a one-way ANOVA followed by Tukey’s multiple comparison test (*<0.05). N = 5, where each “n” represents 5–7 pooled pituitaries per treatment group)
(TIF)
