# Supplementary figures and images for: Maximal Expression of Foxl2 in Pituitary Gonadotropes Requires Ovarian Hormones
Source: PLoS One. 2015 May 8;10(5):e0126527. doi: 10.1371/journal.pone.0126527 (PMC4425675; doi:10.1371/journal.pone.0126527)

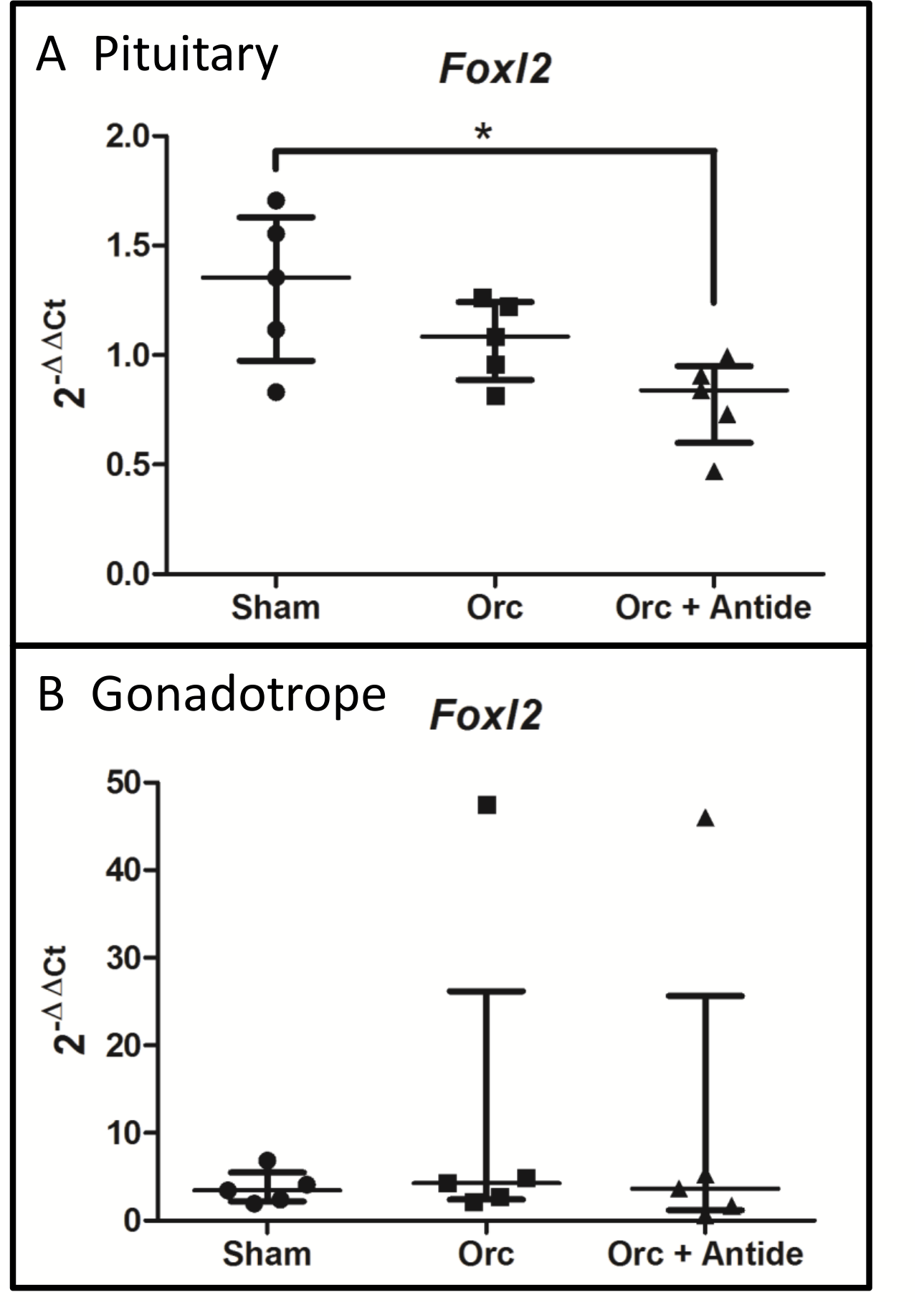

Supplement: S1 Fig — Male mice were either sham operated or orchiectomized and then treated with vehicle or Antide every other day for 10 days. Pituitaries were harvested and gonadotrope specific transcripts isolated. Real time PCR was performed with primers specific to Foxl2 and Rpl19, used for normalization. 2-ΔΔCt values were calculated using total RNA from LβT2 cells as the calibrator. The median with interquartile range is shown for A.) total pituitary Foxl2 levels and B.) gonadotrope Foxl2 levels. Statistical analysis was performed with a one-way ANOVA followed by Tukey’s multiple comparison test (*<0.05). N = 5, where each “n” represents 5–7 pooled pituitaries per treatment group) (TIF) [file pone.0126527.s001.tif]
